# Association Between Rheumatoid Arthritis and Serum Vitamin D Levels

**DOI:** 10.7759/cureus.18255

**Published:** 2021-09-24

**Authors:** Naintara Sukharani, Kapeel Dev, FNU Rahul, Pinky Bai, Azka Ali, FNU Avinash, Yasir Kammawal, Narindar Kumar, Amber Rizwan

**Affiliations:** 1 Internal Medicine, Peoples University of Medical & Health Sciences for Women, Nawabshah, PAK; 2 Internal Medicine, Ghulam Muhammad Mahar Medical College, Sukkur, PAK; 3 Internal Medicine, Jinnah Sindh Medical University, Karachi, PAK; 4 Internal Medicine, King Edward Medical University, Lahore, PAK; 5 Anatomical and Clinical Pathology, University of Arizona College of Medicine, Tucson, USA; 6 Internal Medicine, Bhitai Dental and Medical College, Mirpurkhas, PAK; 7 Family Medicine, Jinnah Post Graduate Medical Center, Karachi, PAK

**Keywords:** rheumatoid factor, autoimmune, rheumatoid arthritis, vitamin-d deficiency, vitamin d

## Abstract

Introduction

Vitamin D is responsible for regulating innate and adaptive immune responses and for boosting the immune system; hence, a decline in its levels results in autoimmunity. Current studies have linked the deficiency of vitamin D to different autoimmune diseases, including rheumatoid arthritis (RA). In this study, we will determine the association between vitamin D level and RA.

Methods

This is a case-control study, conducted in a tertiary care hospital in Pakistan from January 2021 to May 2021. Three hundred patients with a confirmed recent diagnosis of RA were enrolled as the study group. Another 300 participants without RA, matched for age and gender, were enrolled in the study as a control group. RA was diagnosed on the basis of clinical symptoms, radiological features on X-ray, and anti-citrullinated protein levels of more than 20 u/mL.

Results

The mean vitamin D level in participants with RA was significantly lower than in the placebo group (30.18 ± 6.27 vs. 38.29 ± 7.98; p-value: <0.0001). The mean vitamin D level in participants with positive RF patients was significantly lower compared to rheumatoid factor (RF)-negative RA patients (29.21 ± 5.16 vs. 32.26 ± 7.02; p-value: <0.0001). There were more participants with hypovitaminosis D in RF-positive participants as compared to RF negative (88.6% vs. 44.3%; p-value: 0.00001).

Conclusion

There is a high prevalence of vitamin D deficiency in patients with RA and there is a link with disease severity. Therefore, a high index of suspicion is required while evaluating the at-risk patients, especially women, with complaints of vitamin D deficiency. Vitamin D supplementation may be needed for the prevention or avoidance of the progression of the disease.

## Introduction

Rheumatoid arthritis (RA) is primarily a chronic inflammatory debilitating disease of the lining of the synovial joints [1]. Not only does it involve the joints, but it is a condition with multisystem involvement. The prevalence of RA is around 0.5-1% of the world’s population [2]. The pathogenesis of RA is an autoimmune and inflammatory disease, which includes the pathologic activation of osteoclasts, B- and T-cells, fibroblasts, chondrocytes, dendritic cells, and proteolytic enzymes, which result in damage to cartilage, bone, and tendons [3]. Moreover, the activation of the immune system leads to severe complications, causing systemic and extra-articular RA manifestations [3].

Immune cells express vitamin D receptors. Vitamin D is responsible for regulating the innate and adaptive immune responses and for boosting the immune system; a decline in its levels results in autoimmunity [4]. Several autoimmune diseases are linked to vitamin D, including insulin-dependent diabetes mellitus, multiple sclerosis, inflammatory bowel disease, systemic lupus erythematosus, and RA [5]. Limited data are available studying the association of vitamin D levels with RA, particularly in Pakistan. In this study, we will determine the link between vitamin D levels and RA in the local population.

## Materials and methods

This is a case-control study, conducted in a tertiary care hospital in Pakistan from January 2021 to May 2021. Three hundred (300) patients with a confirmed recent diagnosis of RA were enrolled as the study group from the outpatient department. RA was diagnosed on the basis of clinical symptoms, radiological features on X-ray, and anti-citrullinated protein levels of more than 20 u/mL. Another 300 participants without RA, matched for age and gender, were enrolled in the study as a control group. The control group was taken from patients' relatives or attendants who accompanied them to the hospital. Participants taking vitamin D supplements, as informed by them, were excluded from the study. Patients were enrolled via consecutive convenient non-probability sampling. The entire procedure was explained to each participant, and their consent was taken. Ethical review board approval was taken from the People's University of Medical and Health Sciences for Women (IRB number: PUMHS/IRB/2021-07) before the enrollment of patients.

After enrollment, data were collected for variables such as gender, age, smoking history, rheumatoid factor (RF), and comorbidities like diabetes, hypertension, and asthma. Phlebotomy was done to collect 5 mL of blood via the cubital vein and was sent to the laboratory to assess vitamin D levels. Patients with vitamin D levels of less than 30 ng/ml were classified as hypovitaminosis D [6].

Statistical analysis was done using SPSS v. 22.0 (IBM Corporation, Armonk, New York). Normality was assessed via a Q-Q plot. Continuous variables, including age and mean vitamin D levels, were presented as mean and standard deviation. Categorical variables, including gender, comorbidities, were presented as percentages and frequencies. T-test was applied to compare the mean age and chi-square was applied to compare categorical data between the two groups. A p-value of less than 0.05 represented a significant difference between the case and control groups and the null hypothesis was void.

## Results

The mean age of participants with RA was 45 ± 11 years while that of the control group was 47 ± 11. All parameters, including gender distribution and comorbidities, were compared between the two groups, and no significant difference was found (Table 1).

**Table 1:** Comparison of the characteristics of participants of both groups BMI: body mass index, kg/m^2^: kilograms per square meter, NS: nonsignificant

Characteristics	Study group (n=300)	Control group (n=300)	p-value
Age in years (Mean ± SD)	45 ± 11	47 ± 11	NS
Male (%)	112 (37.3%)	119 (39.6%)	NS
BMI greater than 30 kg/m^2^ (%)	52 (17.3%)	54 (18.0%)	NS
Diabetes (%)	72 (24.0%)	77 (25.6%)	NS
Hypertension (%)	82 (27.3%)	79 (26.3%)	NS
Current smokers (%)	57 (19.0%)	55 (18.3%)	NS

The mean vitamin D level in participants with RA was significantly lower compared to the control group (30.18 ± 6.27 vs. 38.29 ± 7.98; p-value: <0.0001). There were more participants with hypovitaminosis D in RA as compared to the control group (77.0% vs. 45.6%; p-value: <0.0001) (Table 2).

**Table 2:** Comparison of vitamin D levels in study and control group ng/mL: nanograms per milliliters, RA: rheumatoid arthritis

Characteristics	Study group (n= 300)	Control group (n= 300)	p-value
Mean vitamin D levels (ng/mL)	30.18 ± 6.27	38.29 ± 7.98	<0.0001
Hypovitaminosis D (%)	231 (77.0%)	137 (45.6%)	<0.0001

In the RA group, 221 (73.6%) participants were RF positive. Mean vitamin D level in participants with positive RF patients was significantly lower compared to RF negative RA patients (29.21 ± 5.16 vs. 32.26 ± 7.02; p-value: <0.0001). There were more participants with hypovitaminosis D in RF positive participants compared to the RF negative (88.6% vs. 44.3%; p-value: <0.0001) (Table 3).

**Table 3:** Comparison of vitamin D levels in association with rheumatoid factor ng/mL: nanograms per milliliters, RA: rheumatoid arthritis, RF: rheumatoid factor

Characteristics	RA patients with positive RF (n= 221)	RF patients with negative RF (n= 79)	p-value
Mean vitamin D level (ng/mL)	29.21 ± 5.16	33.26 ± 7.02	<0.0001
Hypovitaminosis (%)	196 (88.6%)	35 (44.3%)	<0.0001

## Discussion

The results of our study demonstrated that RA patients had lower levels of vitamin D. Moreover, the mean levels of vitamin D were lower in patients with RF positivity than in RF-negative RA participants. Similarly, RF-positive RA patients were reported to have hypovitaminosis more frequently than RF-negative RA patients.

In accordance with the results of our study, a cross-sectional study by Atwa et al. reported that vitamin D deficiency was significantly more prevalent in RA patients [6]. Another retrospective study by Haque et al. suggested that 61% of the RA patients were deficient in vitamin D [7]. A prospective cohort study on 29,368 normal women was conducted to study the correlation between vitamin D and RA activity. Their diet was supplemented with vitamin D. The cohort reported only 152 cases of RA in these women over a span of the 11-year follow-up, suggesting a significant reduction in the risk of RA with increased intake of vitamin D supplements [8].

Literature has time and again stated an integral role of vitamin D in the pathogenesis of autoimmune disorders, including RA [9]. RA is believed to be induced by the interaction of environmental elements in patients with genetic vulnerability [10-12], potentially causing impaired functioning of innate and adaptive immunity, disturbing the equilibrium between autoimmunity and endurance [13]. Even though smoking is considered one of the main environmental risk factors of RA, vitamin D is also a potential factor [9]. On the other hand, since RA restricts mobility, affected patients restrict their outdoor activities [14]. This, in turn, limits their sun exposure, which is the main source of vitamin D. Further deficiency of this vitamin could also exacerbate the disease.

Generally, a couple of studies have evaluated the effect of RA treatment regimens on the evident converse connection between serum 25-hydroxyvitamin D3 (25-OHD3) and RA disease activity [15-17]. Treatment for RA is pointed towards decreasing disease activity, maximizing joint function, and, along these lines, keeping a check on serum 25-OHD3 levels. The inception of treatment early in the disease could cover the effects of vitamin D deficiency, causing a decline in the proportion of disease activity. Since the 1990s, an early and vigorous way to deal with the treatment of new RA has been broadly utilized to amplify the chances of inducing remission [18]. Today, this procedure is completely coordinated in clinical practice, yet its application with regards to low vitamin D and vitamin D substitution for early treatment, recently analyzed in the RA patients, is so far indistinct.

Keeping in view the above findings, our study highlights the importance of vitamin D in RA. However, there are a few limitations as well. First, the study was conducted in a single institute, the sample size was limited and less diverse. Secondly, since it was a case-control study, it was not possible to establish the causal relationship between the two. Future multi-centered prospective research studies with larger sample sizes and diversity are required to confirm the findings of our study. Moreover, studies assessing the dosage of vitamin D supplementation are also required to explore better treatment strategies.

## Conclusions

Evidence shows a high prevalence of vitamin D deficiency in patients with RA and its link with disease severity. It may be a causative factor in its development or progression. Therefore, a high index of suspicion is required while evaluating the at-risk patients, especially women, with complaints of vitamin D deficiency. Vitamin D supplementation may be needed for the prevention of RA. In patients with RA, it is important to keep a check on vitamin D levels and its supplementation, as required, to avoid the progression of the disease.
